# Power of linkage analysis using traits generated from simulated longitudinal data of the Framingham Heart Study

**DOI:** 10.1186/1471-2156-4-S1-S28

**Published:** 2003-12-31

**Authors:** Dai Wang, Xiaohui Li, Ying-Chao Lin, Kai Yang, Xiuqing Guo, Huiying Yang

**Affiliations:** 1 Division of Medical Genetics, Medical Genetics Birth Defect Center, Cedars-Sinai Research Institute, Cedars-Sinai Medical Center, 8700 Beverly Boulevard, Los Angeles, California, 90048 USA; 2 University of California, Los Angeles, California, 90095 USA

## Abstract

The Framingham Heart Study is a very successful longitudinal research for cardiovascular diseases. The completion of a 10-cM genome scan in Framingham families provided an opportunity to evaluate linkage using longitudinal data. Several descriptive traits based on simulated longitudinal data from the Genetic Analysis Workshop 13 (GAW13) were generated, and linkage analyses were performed for these traits. We compared the power of detecting linkage for baseline and slope genes in the simulated data of GAW13 using these traits. We found that using longitudinal traits based on multiple follow-ups may not be more powerful than using cross-sectional traits for genetic linkage analysis.

## Background

In the past 50 years, the Framingham Heart Study has been a very successful longitudinal research study of cardiovascular diseases. Over the years, many of the major cardiovascular disease risk factors, i.e., high blood pressure, high blood cholesterol, smoking, obesity, diabetes, and physical inactivity, have been identified through careful monitoring of the Framingham Heart Study population. A large amount of valuable information on the effects of related factors, such as blood triglyceride and HDL cholesterol levels, age, gender, and psychosocial issues, has been collected. In the mid-1990s, a genome scan was conducted for 330 pedigrees selected from the Framingham Heart Study. This raised a series of interesting questions such as: how to use the longitudinal phenotypic data in linkage analysis? Would longitudinal data provide more power for demonstrating linkage?

Longitudinal phenotypic data contain information not only on trait values at a specific time point, but also on the progression of a trait over time. In addition to identifying genes responsible for cross-sectional trait values, longitudinal data also provide the possibility of identifying genes related to the progression of a trait across time. The progression of a complex trait may depend on many environmental factors and gene × environment interactions. Therefore, identifying genes related to the progression of a complex trait may help us study the environmental factors and the gene × environment interactions involved. So far, three types of descriptive traits have been used for linkage analysis of longitudinal data: single visit value [1], within-subject mean across the visits [2-4], and the changes between two visits [5]. The simulated data of the Genetic Analysis Workshop 13 (GAW13) (100 replicates) provided us with an opportunity to evaluate the power of linkage analysis for identifying loci responsible for baseline or slope variations using these descriptive traits. In addition to the above three types of traits, we introduced a new descriptive trait for linkage analysis: within-subject slope of the trait. We compared the power of linkage analysis using these descriptive traits for identifying baseline and slope genes in the simulated data of GAW13.

## Methods

### Data Structure

The simulated data of GAW13 were generated based on the real data scenario of the Framingham Heart Study. The family structure was composed of 330 pedigrees selected for the genome scan of the Framingham Heart Study. The pedigrees consisted of 4692 subjects, of whom 2885 had participated in the Framingham Heart Study. Among the 2885 participants, there were 3041 parent-offspring pairs, 2796 sib-pairs, 2107 avuncular pairs, 183 grandparent-grandchild pairs, and 1595 first cousin pairs. The same family structure was used for simulating all 100 replicates. For each of the 100 replicates, a total of 399 microsatellite markers on the 22 autosomal chromosomes were simulated using the allele frequencies of the markers from the Framingham Heart Study data. Each replicate contained longitudinal data for two cohorts, with data collection on each cohort starting about 30 years apart. The first cohort was examined 21 times at 2-year intervals, while the second cohort was examined 5 times with an 8-year interval between the first two exams and 4-year intervals between subsequent exams. Both completed data and data with missing values were provided for analysis. For simplicity, we used complete genotype and phenotype data for our analysis.

### Phenotypes

GAW13 simulated data provided phenotypic data on age, sex, height, weight, cholesterol, blood pressure, glucose, and various other traits. We focused our analysis on cholesterol and its related covariates, such as sex, age, body mass index (BMI), and triglycerides (TG). There were 21 visits for the first cohort, and only five visits for the second cohort. In order to take advantage of the longitudinal nature of the phenotypic data, we constructed several descriptive traits for linkage analysis. To make the variance of these descriptive traits as comparable as possible for both cohorts, visit 1, 5, 7, 9, and 11 were selected from the first cohort, corresponding to the time intervals of the visits in the second cohort, to generate the descriptive traits. Furthermore, only subjects with cholesterol data at all five visits were used in the analysis for both cohorts. The following traits were generated for linkage analysis.

1) **CHOL1**: the total cholesterol level at the first visit. It contains genetics effects mainly from the baseline genes.

2) **CHANGE**: the change of the total cholesterol level over 20 years. For Cohort 1, it was the change of the cholesterol level from visit 1 to visit 11; for Cohort 2, it was the change of the cholesterol level from visit 1 to visit 5. It contains the genetic effects mainly from the slope genes.

3) **MEAN**: the within-subject mean of the total cholesterol level across the five visits. It contains the genetic effects from both the baseline and the slope genes.

4) **SLOPE**: the within-subject slope of the total cholesterol level of each individual for the five visits regressed on age. It contains the genetic effects mainly from the slope genes.

Among the four descriptive traits we considered here, CHOL1 was based on the data at the first visit only, CHANGE was generated based on the data from two out of five visits, and MEAN and SLOPE were generated based on data from all five visits.

### Statistical analysis

Two-point sib-pair linkage analysis was conducted using SIBPAL in S.A.G.E. 3.1 [6]. Sex, age, BMI, and log(TG) were included in the analysis as covariates for CHOL1, CHANGE, and MEAN. Sex, BMI, and log(TG) were included in the analysis as covariates for SLOPE.

Multipoint sib-pair linkage analysis was conducted with MAPMAKER/SIBS in GENEHUNTER2.1_r3 Beta program package [7,8]. The residuals after the adjustment for sex, age, BMI, and log(TG) were used in the analysis for CHOL1, CHANGE, and MEAN. The residuals after the adjustment for sex, BMI, and log(TG) were used in the analysis for SLOPE. Families too large for GENEHUNTER to analyze were divided into smaller families.

## Results and Discussion

According to the answer key distributed by GAW13, Gb30-Gb33 and Gs7-Gs9 were the seven genes influencing cholesterol levels directly. Among the seven genes, Gb30-Gb33 were baseline genes and Gs7-Gs9 were slope genes. Both two-point and multipoint sib-pair linkage analyses were carried out for the four descriptive traits on the chromosomes containing the baseline and the slope genes for total cholesterol level, that is, chromosomes 1, 3, 7, 11, 13, 15, and 21. In order to evaluate the false-positive rate, we also analyzed chromosome 2, which does not have any trait locus.

Two-point linkage analysis using SIBPAL produced a *p*-value for each marker locus. For each trait locus, we took the smallest *p*-value out of the four markers around the trait locus, which was equivalent to a 30-cM range, as the significance level for that trait locus. We counted the number of times the smallest *p*-value among the four markers was less than 0.0125 (0.05/4, Bonferroni correction) out of the 100 replicates as the power of detecting linkage. Table 1 shows the power of detecting linkage with the four descriptive traits generated from the longitudinal data using SIBPAL. For chromosome 2, we counted the number for every four adjacent markers. The average number across chromosome 2 was then taken as the false-positive rate.

A multipoint LOD score was calculated for each locus by using MAPMAKER/SIBS. For each trait locus, again, we considered four markers around it. We counted the number of times that the largest LOD score among the four markers was greater than 1.67 (corresponding asymptotically to a 0.05 significance level) as evidence for suggestive linkage. Table 2 shows the power of detecting linkage with the four descriptive traits using MAPMAKER/SIBS. The false-positive rate in Table 2 was calculated in a similar way as in Table 1.

From Table 1 and Table 2, we observed that the false positive rates for both two-point and multipoint linkage analyses were within a reasonable range (1.0~7.1%). As expected, different descriptive traits had different power for detecting linkage of different genes. CHOL1 had a moderate power to detect linkage for the baseline genes (~60% for Gb30), but not for the slope genes (power <21% for Gs7). CHANGE and SLOPE had approximately 80% power for detecting linkage for the slope gene Gs7, but no power for detecting the baseline genes (<10% for Gb30-G33). MEAN had moderate power to detect linkage for both baseline (49% to 63% for Gb30) and slope genes (62% to 80% for Gs7).

According to data description of the simulated data of GAW13, the contributions of the four baseline genes (Gb30-Gb33) to the baseline variance of total cholesterol level were 0.20, 0.15, 0.1, and 0.05, respectively. In our linkage analysis results, Gb30 was detected with the highest power while Gb33 was detected with the lowest power. This demonstrated that genes with higher contribution to the trait variance could be detected with higher power. Among the three slope genes, Gs9 contributed to the slope of females only and its contribution to the variance of the slope is very low (~0.03). Here, we actually did not have any power to detect this gene using either two-point or multipoint linkage analyses. For the other two slope genes, the power for detecting Gs7 was higher than that for Gs8, which was also consistent with their contributions to the variance of the slope (0.36 for Gs7 and 0.08 for Gs8).

For the two traits containing the genetic effects from baseline genes, CHOL1 and MEAN, the total cholesterol level at the first visit (CHOL1) had a similar or sometimes even higher power to identify baseline genes compared to MEAN. Thus CHOL1 is an acceptable trait for detecting linkage for baseline genes, especially when genetic effects are relatively large. Two longitudinal genetic studies [9,10] also showed that heritability estimates were high at time point 1 and remained stable across time.

CHANGE, MEAN, and SLOPE were the three traits containing genetic effects from slope genes. For a slope gene with large effect, e.g., Gs7, SLOPE is the most powerful trait and MEAN is the least powerful trait from both two-point and multipoint analyses. However, improvement in power from using SLOPE to using CHANGE was limited (from 0.79 to 0.80 in two-point analysis and from 0.83 to 0.87 in multipoint analysis) while SLOPE used much more data than CHANGE (five visits vs. two visits). For a slope gene with relatively small effect, e.g., Gs8, power was low for both analytic methods.

In summary, in comparison with descriptive traits generated from multiple longitudinal data points (such as MEAN and SLOPE), CHOL1 had a similar or even higher power for detecting baseline genes as MEAN, and CHANGE had a similar power for detecting slope genes as SLOPE. The possible explanation for this is that genetic effects are relatively stable in an individual's lifetime, at least in this simulated situation. However, MEAN and SLOPE used the data of all five visits while CHOL1 and CHANGE required data of one or two visits only. Therefore, conducting a longitudinal analysis with multiple follow-ups may not be an effective way to identify susceptibility genes responsible for either baseline or change over time.

In the simulated data of GAW13, environmental factors did not play an important role in total cholesterol levels. However, in reality, environmental exposures, medications, and gene × environment interactions may play an important role in determining an individual's cholesterol levels, as well as many other complex traits such as DBP and SBP. Under such circumstances, the usefulness of a longitudinal study design with multiple visits should be further explored.

## Figures and Tables

**Table 1:** Power for detecting linkage (%) with four descriptive traits using SIBPAL

	Baseline Genes				Slope Genes			
			
	Gb30	Gb31	Gb32	Gb33	Gs7	Gs8	Gs9	False Positive
CHOL1	63	52	29	14	21	10	4	3.7
CHANGE	3	5	7	4	79	13	6	6.0
MEAN	49	41	26	12	62	12	4	3.6
SLOPE	3	4	9	6	80	16	6	5.9

**Table 2:** Power for detecting linkage (%) with four descriptive traits using MAPMAKER/SIBS

	Baseline Genes				Slope Genes			
			
	Gb30	Gb31	Gb32	Gb33	Gs7	Gs8	Gs9	False Positive
CHOL1	64	32	8	4	11	6	0	1.0
CHANGE	2	5	2	5	83	15	6	4.9
MEAN	63	40	23	10	80	20	4	5.0
SLOPE	6	6	4	7	87	14	7	7.1
